# Factors Influencing COVID-19 Vaccine Uptake among Spanish-Speaking Pregnant People

**DOI:** 10.3390/vaccines11111726

**Published:** 2023-11-17

**Authors:** Magali Sanchez, Iveliz Martel, Elizabeth Cox, Isabelle Crary, Carly Baxter, Emma Every, Jeff Munson, Simone Stapley, Alex Stonehill, Kristina M. Adams Waldorf

**Affiliations:** 1 Department of Epidemiology, School of Public Health, University of Washington, Seattle, WA 98195, USA; magalis@uw.edu; 2 Department of Drama, University of Washington, Seattle, WA 98195, USA; liztelmar@gmail.com; 3 Department of Health Systems and Population Health, School of Public Health, University of Washington, Seattle, WA 98195, USA; 4 School of Medicine, University of Washington, Seattle, WA 98195, USA; 5 Department of Psychiatry, University of Washington, Seattle, WA 98195, USA; 6 Department of Communication, University of Washington, Seattle, WA 98195, USA; 7 Department of Obstetrics and Gynecology, University of Washington, Seattle, WA 98195, USA; 8 Department of Global Health, University of Washington, Seattle, WA 98195, USA

**Keywords:** pregnancy, vaccine, vaccine hesitancy, COVID-19, social media, rural medicine, Spanish, Hispanic, Latina, maternal health

## Abstract

The coronavirus disease 2019 (COVID-19) pandemic exposed the vulnerability of pregnant women to excess morbidity and mortality, as well as the disproportionate disease burden in certain racial, ethnic, and sociodemographic groups. Vaccine hesitancy represents a major threat to public health, and crafting messages that reach vulnerable groups and address their intersectionality remains a weakness for pandemic preparedness. We sought to investigate factors that influenced vaccine acceptance and social media ad response in a mixed-methods study of Spanish-speaking women living in the rural Western United States who were pregnant or recently pregnant between November 2022 and June 2023. Direct interviews were translated, transcribed, and coded, while the ad ratings were analyzed using linear mixed models. Participants most favorably rated ads that featured doctors and text-heavy content describing benefits of vaccination. Qualitative data illustrated how information from trusted medical providers along with generational and cultural history of vaccine acceptance positively impacted perspectives on vaccination. Immigration status had varying influences on vaccination perspectives. Future vaccination campaigns targeting Spanish-speaking pregnant individuals in rural communities should use medical providers as ad messengers and dispel fears that vaccine acceptance may lead to problems with immigration status.

## 1. Introduction

The coronavirus disease 2019 (COVID-19) created a pandemic in 2020 as a novel coronavirus spread through respiratory droplets from coughing and sneezing. The mortality rate from COVID-19 was considerable, especially within vulnerable groups such as older and immunocompromised adults. Pregnant women had higher rates of mortality and morbidity from COVID-19, which mainly occurred in unvaccinated people [1,2,3,4,5,6,7,8,9]. Many factors contributed to high vaccine hesitancy within pregnant populations including vaccine disinformation that implied vaccination negatively impacts fertility and/or fetal development [10,11,12,13,14,15]. Major threats to pandemic preparedness are vaccine hesitancy and the lack of knowledge related to factors driving vaccine acceptance within vulnerable groups and specific sociodemographic and racial/ethnic groups [16,17,18,19,20]. Increasing vaccine uptake in reproductive-aged women and pregnant women will require knowledge of the factors that positively influence vaccine uptake.

More information is needed to determine how public health campaigns can deliver vaccination information to reach vulnerable groups, like pregnant women. Social media has the potential to revolutionize public health messaging by making important health messages accessible to a wide audience quickly. Crafting a successful health information ad campaign on social media requires evidence for who the target audience considers as a trusted messenger for the information and the ad content (e.g., information, fear based) that they prefer to view. Few studies have addressed this important question for pregnant women especially within specific racial, ethnic, and socioeconomic groups reported to be highly vaccine hesitant [10,21]. COVID-19 vaccine uptake was initially slower for people self-identifying as Hispanic or Latine than in many other racial/ethnic groups (e.g., non-Hispanic White, Asian) [22,23,24]. COVID-19 disease burden was also greater in rural versus urban Spanish-speaking Hispanic/Latine communities likely due to lower socioeconomic status, greater housing density, and potentially greater structural burdens to accessing vaccination in rural settings [25,26]. More information is needed about vaccine decision-making that incorporates the diverse identities present within specific racial/ethnic groups.

The objective of our mixed-methods study was to investigate factors informing vaccine decision-making in Spanish-speaking pregnant or recently pregnant women in the Western U.S. through qualitative interviews, as well as to assess the efficacy of social media ads promoting COVID-19 vaccination during pregnancy through quantitative analysis. We hypothesized that Hispanic/Latine women would rate faith-based messengers and ads discussing protection of the fetus and family as the highest when compared to other messengers (i.e., doctor, elder, peer) and content types (i.e., text heavy, social proof, information on negative outcomes). The goal of the study was to address the literature gap relating to determinants of vaccine hesitancy in this unique population while also providing strategies to combat this vaccine hesitancy through evaluation of social media ads. Greater information on vaccine decision-making and social media ad engagement within specific vaccine hesitant groups can help inform culturally appropriate public health campaigns geared toward improving maternal health or saving lives among Hispanic/Latine women during a pandemic [27,28].

## 2. Materials and Methods

### 2.1. Study Design and Ethics Approval

This study utilized a mixed-methods research design to evaluate vaccine hesitancy and vaccine-related knowledge gaps among pregnant Hispanic individuals along with the efficacy of social media advertisements in countering vaccine hesitancy. Qualitative interviews provided novel insight into factors influencing vaccine hesitancy during pregnancy that could not otherwise be elicited for quantitative data, while quantitative data allowed for evaluation of the impact of social media advertisements within this population. Qualitative data also served to augment, support, and explain results uncovered in the quantitative data collection. The University of Washington IRB granted ethics approval to conduct this study. Informed consent was collected from all participants prior to conducting interviews.

### 2.2. Participants and Procedures

We interviewed 30 Spanish-speaking participants who were currently pregnant or had given birth within 6 months of the interview between November 2022 and June 2023 and lived in rural counties in Arizona, California, Idaho, Nevada, Oregon, Utah, and Washington. Participants were recruited into the study through social media ads on Facebook and Instagram. These ads were developed to target Spanish-speaking women between the ages of 18 and 40 who were pregnant or who had given birth within the last 6 months and who resided in counties with fewer than 50,000 people. The target audience also included individuals who had shown interest in pregnancy-related products such as diapers, baby powder, formula, etc. The target audience was estimated to be 1,000,000–1,200,000 people, and the ads received 2344 link clicks. Participants filled out a REDCap contact survey linked to the ad. Participants confirmed they met study criteria and filled out their contact information. We confirmed the rural status of participants by cross-referencing their zip codes with a tool provided by the Health Resources and Services Administration. Eligible participants were contacted at least 3 times via phone or email and scheduled for an interview over Zoom. Participants were interviewed by a native Spanish speaker using a standardized interview guide (Table S1). The survey closed once 30 participants had been interviewed.

### 2.3. Measures and Instruments

Demographic information, including questions pertaining to race, income, education, employment, marital status, political affiliation, religious affiliation, and COVID-19 vaccination status, was collected through a participant-completed online survey. Following completion of the survey, respondents completed a structured 45–60 min online video interview through Zoom. The interview included 24 open-ended questions pertaining to perspectives on the COVID-19 vaccines, sources of COVID-19 vaccine hesitancy, and use of social media, among others (Table S1). The interview guide was initially developed for our initial study on vaccine hesitancy, which was modeled on principles of the 3C Model on Vaccine Hesitancy and The Health Belief Model [29,30]. Questions were further refined following feedback from key informant interviews. Translated interview questions were pilot tested and validated by bilingual individuals. Following the interview, participants were shown four advertisements relating to COVID-19 vaccine promotion which they then evaluated using a closed-ended Likert scale question and one open-ended question pertaining to willingness to receive the COVID-19 vaccine following viewing of the advertisement. Interview participants received a USD 50 Amazon gift card upon completion of both the survey and interview.

### 2.4. Ad Design

To evaluate the effect and efficacy of social media advertisements in the Hispanic population, we designed 16 unique ads promoting the COVID-19 vaccine. Each advertisement featured a specific messenger (peer, doctor, elder, faith leader) and content type (appeal to protect, text heavy, social proof, information on negative outcomes, or activation). Design of the advertisements and marketing principles were similar to our previously published methods in an English-speaking rural population [21], but the ads were designed specifically for a Hispanic pregnant population in Spanish.

In the Zoom interview, the ad order shown to the participants was randomized based on messenger type but not content type. Participants were asked to respond with their initial reaction to the advertisement, which was evaluated qualitatively. Participants then were asked to use a five-point Likert scale (from “Strongly Agree” to “Strongly Disagree”) to assess whether they would be more likely to receive the COVID-19 vaccine or booster during pregnancy after interacting with the ad content.

### 2.5. Data Analysis

This mixed-methods study included both qualitative and quantitative data collection and analysis, with qualitative data being derived from the open-ended interview questions and quantitative data originating from the pre-interview survey and Likert scale reaction to the advertisements. The quantitative analysis was focused on evaluating the effects of specific ad messengers and content type on ad ratings with each participant viewing and rating four different ads. Statistical analysis employed linear mixed models using the R packages “lme4” and “lmeTest”. Separate models were evaluated for each independent variable as there was an uneven distribution of the combination of messenger and content types with some messenger/content combinations not being represented as they were unlikely (e.g., faith-based messenger with negative outcomes message). “Doctor” and “Text-Heavy” were set as the reference categories for the messenger and content variables, respectively, as these options were rated most favorably.

Prior to the qualitative analysis, interviews were transcribed and translated into English. Next, transcripts were blindly coded using Dedoose 9.0 (thematic codebook in Table S2) separately by two evaluators. Quotations were extracted for each theme and combined into a single document. Coders met to discuss themes, evaluate areas of the transcript when codes did not align, and complete the final analysis.

## 3. Results

### 3.1. Participants

In this study, thirty Spanish-speaking pregnant or recently pregnant women were interviewed individually by a native Spanish speaker (Table 1). The majority were currently pregnant and vaccinated for COVID-19 at the time of the interview. Most reported Catholic as their religious affiliation.

### 3.2. Quantitative Analysis of Social Media Ad Reactions Promoting Vaccination

**Figure 1:**
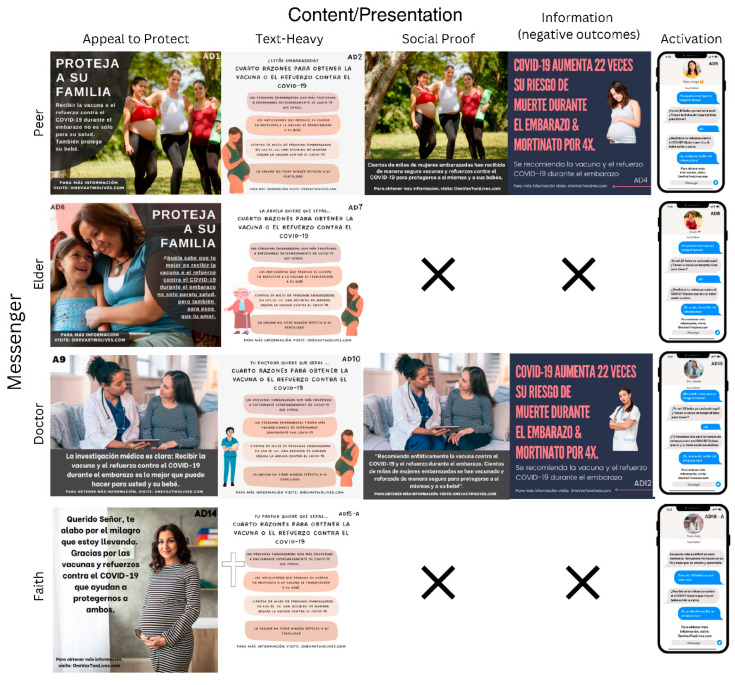
Combinations of messengers and content themes in social media ads.

We developed 16 sample social media ads that tested four messengers (peer, doctor, elder, faith leader) and five types of content (activation, social proof, text heavy, appeal to protect) to evaluate how participants viewed different messengers and content and their various combinations. Figure 1 illustrates the ad combinations, and Figures S1–S4 contain larger versions with readable text. Ten ad sets were developed based on the 16 ads to randomize combinations of messenger and content type (Table 2). After participants were shown an ad, researchers asked one qualitative and one quantitative question. The qualitative question focused on assessing what the participant liked and disliked about the ad, while the quantitative question evaluated each ad’s impact on the participant’s likelihood to receive a COVID-19 vaccine using a Likert scale (1–5 score).

Figure 1 demonstrates social media ads classified by messenger and content type. When possible, messenger and content were kept similar across ads to allow for comparisons. However, certain combinations were not realistic and, therefore, not created.

**Figure 2:**
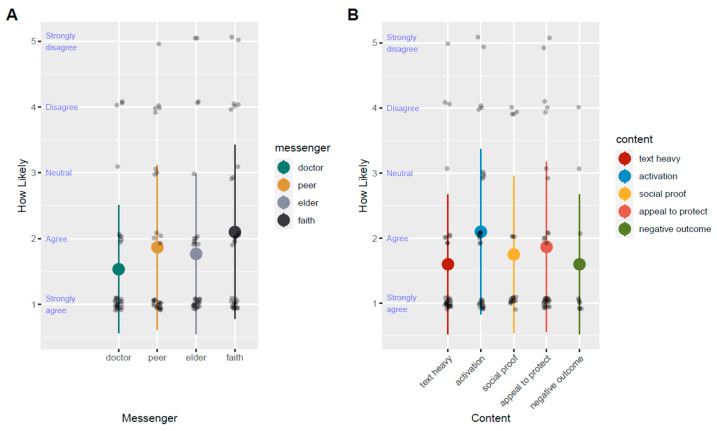
Likelihood of becoming vaccinated or boosted after seeing an ad by messengers (**A**) and content (**B**). The small gray dots indicate the individual participant scores. The large circles indicate the mean in each category.

We used a mixed-effects model to analyze social media ad responses on a Likert scale. First, we determined the messenger that received the lowest ratings indicating the highest likelihood to become vaccinated after seeing the ad (Figure 2A). Doctor was selected as the reference category as it was the most favorably ranked messenger. Only the faith leader messenger was rated significantly less favorably than ads depicting the doctor messenger (*p* = 0.005, Table 3 and Table S3). Next, we investigated the participants’ preferences for ad content (Figure 2B). The most favorably ranked content type, text-heavy ads, was set as the reference category. Among the four content types, activation-based content, designed to motivate participants to get vaccinated, was the only content type rated significantly less favorably than negative-outcomes-based ads (*p*-value = 0.01, Table 4 and Table S4).

This figure illustrates the self-rated likelihood that a participant might receive a COVID-19 vaccine after seeing an ad with either a specific messenger (A) or content type (B). A low rating indicated “strong agreement” that a participant would be more likely to be vaccinated after seeing the ad. A high rating indicates a low likelihood of becoming vaccinated after seeing the ad.

### 3.3. Qualitative Analysis of Interview Themes and Ad Reactions

Four key themes emerged with 11 sub-themes through the qualitative analysis of the interviews (Table 5 and Table S5). Key themes included doctors as a trusted messenger and their importance in vaccine promotion, immigration status, generational history of vaccine acceptance through a maternal figure, and targeted messages on vaccination benefits for protecting oneself and the fetus. Reactions to social media ads were included to support interview themes.

#### 3.3.1. The Doctor as a Trusted Messenger and Their Importance in Promoting Vaccination

Ads with a doctor messenger were rated the most likely to inspire vaccination, based on the participants’ respect for their extensive education and training. One participant stated, “*I do trust what health providers tell me. I feel that they are educated people who have experience. They are kept informed … they update their knowledge. And, therefore, I trust in what they tell me.*” Another participant said, “*My doctor convinced me … she told me that the symptoms of COVID-19 were going to be less severe and that while I was pregnant, my baby could have a little of the benefits pass on to him.*” As trusted sources of information, medical providers can effectively address concerns about vaccine side effects and possible harmful effects to the fetus, reassuring expecting mothers that the vaccine is safe in pregnancy and will ultimately protect them and their fetus. One participant needed validation from her provider before receiving the vaccine and stated, “*I was already going in [to the clinic] with the mentality that [the doctor] is going to tell me that it is fine, but I am not going to do it before consulting her.*” Respect and trust in the medical profession to provide information related to their knowledge regarding vaccination in pregnancy were strong themes.

Text-heavy ads reinforcing messages they heard from doctors that explained the vaccine was safe and passed antibodies from mother to fetus were associated with ratings reflecting the greatest likelihood of vaccination after seeing the ad. One participant stated, “*I feel like it [text-heavy ad] makes me want more information, to talk it over with my doctor and be more informed because I feel like I wasn’t given the information that maybe was very important. I like that it explains that it won’t have any effects on fertility*.” Statements within the text-heavy ads debunking myths about the vaccine’s effects on fertility were also valued. Text-heavy ads also made participants reflect on what their providers had not told them about the COVID-19 vaccine. In response to Ad 2 (peer, text-heavy ad), one participant stated: “*If my gynecologist had told me, I would have had the vaccine sooner… I suspected [the statement] about the antibodies because with the tetanus vaccine I already knew that the antibodies are transferred to the fetus, I imagined that, but my gynecologist never told me that*.” Concise, informative text-heavy ads reinforcing information provided by their medical provider that addressed vaccine benefits and myths received the best ratings.

Although participants described trusting their providers, they also described poor quality of care due to insufficient time with their provider, inadequate counseling, and language barriers. One individual stated, “*So, I trust the doctor, I mean, they should be the first source of information, but yes, they should spend a little more time or if they would provide more information, such as, ‘Look, we’re going to do these tests. These exams are for this or [ask] is everything okay?’ But if you don’t ask, they just don’t tell you.*” As rural clinics often had insufficient interpreters for Spanish-speaking patients, some patients tried to learn English in hopes of communicating more effectively with their provider. “*I learned a little English, so then she tries to understand me, and I try to make myself understood by her or she provides me with an interpreter … [but] I personally feel more comfortable when I talk directly to her,*” said another participant. The shorter time spent with patients and lack of available interpreters recurred as themes related to receiving adequate information to make an informed decision to be vaccinated during pregnancy.

Multiple participants reported that medical providers failed to initiate discussions about the COVID-19 vaccine at several visits and even throughout their entire pregnancy. “*Well if they [doctor] tell me, then yes … If they tell me, I need to get vaccinated, then I’ll say yes,*” said one participant who was willing to get vaccinated but did not simply because it was not offered to her by her provider. Another participant was offered a whooping cough vaccine but not the COVID-19 vaccine, indicating a missed opportunity to protect and educate patients. For patients who needed more time to decide whether to be vaccinated, their providers did not always follow up the discussion at subsequent appointments, creating yet another barrier to getting vaccinated. For example, one participant stated, “*At the next appointment with my doctor, I tried to ask him, and it was really close to my due date, and they said, ‘No’ like I had already run out of time [to get the vaccine].*” Physicians, while often a trusted source of information, often did not provide enough encouragement or discussion about the vaccine, which provided a barrier to vaccination in many participants. 

Some participants reported turning to nurses and ancillary staff to help acquire more information especially when their medical provider did not speak Spanish. A participant stated, “*I think we communicated well. However, of course, if there had been another option of a gynecologist that had been in Spanish, then I had taken it without thinking about it.*” Participants reported trusting the ancillary staff, like the nurses or medical assistants, for medical information. Another participant said, “*I asked her [the nurse] a lot … she did a lot of the work that I think the doctor should have done… I probably wouldn’t have known 80% of the stuff about my pregnancy.*” This not only highlights the value of nurses and ancillary staff but brings up the question of who patients turn to when the ancillary staff is not available to help. Other patients reported turning to WIC (Women, Infants, and Children) staff and social workers who were also available to answer the participant’s questions in Spanish and would follow up with participants. 

#### 3.3.2. Immigration Status as an Influential Factor for Vaccine Uptake

The participant’s immigration status acted both positively and negatively on their decision to receive the COVID-19 vaccination during pregnancy. Some individuals seeking citizenship in the United States saw COVID-19 vaccination as a requirement for their medical evaluation for immigration. One stated, “*If you want to pass the evaluation, then you have to do it.*” This requirement promoted even vaccine-hesitant individuals to receive the vaccine. On the other hand, some participants viewed the requirement of going to a clinic or hospital to receive the vaccine as risky because of their immigration status: “*I believe that as an immigrant, sometimes we don’t have easy access or the confidence to go to a hospital. A clinic is very expensive…And there is sometimes the fear of going to give your information to the hospital.*” The vaccine was viewed both as a requirement for immigration medical evaluations and a risk to personal safety and freedom for individuals with concerns about the implications of providing personal information to government entities.

Many individuals reported how their perspectives on vaccination were positively influenced by their culture and experiences in their ancestral country. One individual poignantly stated: “*In Peru … well, people die because there is no hospital, that is, there is no hospital 100 km around, so people die more … so one way to prevent so much death is to get vaccinated. I come from such a culture, one in which we protect ourselves from disease, as much as possible. So, I had never before in my life, ever questioned the effectiveness or credibility of a drug made by professionals by scientists, but here [in the United States] I am in a completely different culture, in which people question, science, the scientific methods. I mean, everything is in question here and honestly, it’s horrible. And that is a cause of a lot of stress, a lot of discomfort for me.*” Her trust in medicine and the barriers for her to access medical care greatly influenced her support of the vaccine. Another participant similarly highlighted the eagerness to become vaccinated in her home country: “*The difference between here and Mexico was that in Mexico, we had to form a line that lasted 2 days to be able to get the vaccine.*” Many participants described growing up with positive views of vaccination in their home country, which was in sharp contrast to what was seen regarding vaccine uptake and enthusiasm in the United States.

#### 3.3.3. Generational History of Vaccine Acceptance through Maternal Figures

Many participants cited their generational history of vaccine acceptance amongst maternal figures in their life as a reason to become vaccinated or to vaccinate their own children. The idea that generations of people before them have trusted vaccines was a source of comfort and security in the safety of a new vaccine. One individual noted, “… *they vaccinate us for everything ever since we are born so then why should I be afraid right now of another vaccine?*” Another person explained that her mother’s decision to vaccinate her as a child was a symbol of care that she can continue to pass onto her children. This person stated, “… *my mom gave me all my vaccines because she loved me, or because she wanted to take care of me… My children are all up-to-date on their vaccinations… for me having all your vaccines is the best proof of love that a parent can give to their children.*” Another stated, “*My mom tells me that I should never miss any [vaccines]… I’ve always seen it as something important for children. And I do not miss it.*” One participant described a maternal figure as a trustful source: “*My grandmother…she was talking to me about vaccines, I think she’s a [trusted] source for me, at least very reliable.*” Maternal support for vaccination was a clear positive influencer for vaccine acceptance. 

#### 3.3.4. Participants Disliked the Use of Faith-Based Social Media Messages Related to Vaccine Uptake

The use of religion in ads was not the most effective messenger in terms of high intent to be vaccinated. Participants who were not religious noted it was not appealing to have an image of a pastor or priest prompting a vaccine. Participants preferred messengers that had information rooted in facts, and, as one participant noted, “*A pastor is going to give this information from a Christian point of view, but if the doctor is based on science and gives me this information, it would be nice*”. Among those who were religious, opinions were mixed. Some participants noted that incorporating a religious messenger might not be effective because beliefs in the vaccine and faith are separate and do not intermix. As one participant stated, “*I feel that it was the opposite of what happened with the pandemic where many people thought that science was against religion … The religious component of this ad would not have motivated me any more or less to receive the booster because I already have my beliefs, so one thing would not have affected the other*”. Others stated the incorporation of a religious messenger would send the opposite message, and, instead, “*It would make me think ‘I’m going to put it in God’s hands’, but it doesn’t motivate you to take the vaccine. It’s more like trusting God than the vaccine itself*”. Lastly, there were some positive responses to religious messengers. One participant stated, “*I am very glad that the person recommending is a pastor because a lot of times I feel that the Latino community is driven by their religious leaders, so if the pastor is recommending it, I think that not only does it urge them to find support in religion and in their faith, but also in the recommendations of their doctors or what the CDC says*”. Though a religious messenger may be emphasized, it should be used in combination with other messengers or facts based on scientific studies. All in all, religious messengers and messaging should not be a priority for broad social media campaigns promoting vaccine uptake but may be appealing in smaller, niche communities that are largely religious.

## 4. Discussion

### 4.1. Summary of Study Findings

This is one of the first studies to investigate social media ad reactions to vaccination in pregnancy in rural Spanish-speaking pregnant or recently pregnant women in the Western U.S. Ads associated with the highest likelihood of vaccination featured doctors as highly trusted messengers and were text-heavy ads describing benefits of vaccination and dispelling myths. Participants tended to dislike ads that featured a faith leader or tried to nudge (activate) the participant to become vaccinated without providing more information. The qualitative data provided unique insight into factors that influence both the decision to get vaccinated and the barriers Spanish-speaking pregnant women face in receiving the vaccine. Overall, participants trusted their medical providers but lack of prompting to get the vaccine by medical providers, along with limitations regarding time and ability to have counseling appropriately translated, provided a barrier to vaccination. Interestingly, immigration status had varying effects on vaccination opinions, with some individuals citing the need for vaccination for immigration evaluation, while others expressed fear that their personal information would be disclosed should they visit a clinic or hospital for vaccination. Participants also expressed how they were often influenced by their own mothers and their support for vaccination and viewed getting their child vaccinated as the role of a good parent. They did not appreciate faith-based messengers. Overall, there is a clear influence of cultural factors, personal relationships, and immigration on vaccine hesitancy, and medical providers can play a strong positive role in counseling and supporting pregnant Spanish-speaking individuals regarding COVID-19 vaccination.

### 4.2. Study Findings in the Context of Literature

Our study findings align with a major pillar of vaccine acceptance in pregnancy, namely the need for sufficient information to make this complex decision. We have recently described the first conceptual model to break down the complex decision-making underlying vaccine uptake in pregnancy which we called the “5-P model” [15]. The 5-P model highlights the following factors as key to decision-making: (1) perceived information sufficiency, (2) protection of pregnancy (harm avoidance), (3) provider–patient relationship, (4) perceived vaccine benefit, and (5) perceived disease susceptibility and severity. Other factors include a history of prior vaccine acceptance, trust in medicine and science, and social determinants of health, like structural barriers (language, access to healthcare). Most of these factors can be targeted in a public health messaging campaign if the trusted messengers and preferred content and platforms are known.

The tension between wanting to get vaccinated to meet requirements for immigration and having concerns about providing identifying information that might jeopardize their stay in the U.S. has been described in prior literature, although not specifically among pregnant Hispanic individuals [23,31]. In fact, undocumented immigrant families have showed greater COVID-19 vaccine acceptance compared to non-immigrant families, despite concerns about providing personal information [23]. These data suggest that vaccination sites not requiring personal information may boost vaccination rates in some Hispanic communities and may save lives of pregnant individuals and others from vulnerable groups.

Our study findings differ from a similar study that our group performed in a different pregnant population to investigate vaccine decision-making in English-speaking women in the Western U.S. [21]. In the English-speaking participants, the peer messenger was the most trusted messenger, and the doctor messenger was associated with ratings indicating a lesser likelihood of vaccination (*p* = 0.06). In contrast to this study, the Spanish-speaking participants preferred ad content that was text and information heavy with a doctor messenger. Trust in doctors to deliver the vaccine message was much higher in the Spanish-speaking pregnant rural population than in English-speaking participants from the same demographic.

### 4.3. Implications for Clinical Care or Public Health Campaigns

The results of our study have actionable implications for public health messaging campaigns, as well as for clinical care. Social media and other communication campaigns targeting Spanish-speaking pregnant patients may benefit from using a doctor messenger. Many Spanish-speaking respondents received little information about the COVID-19 vaccine during pregnancy from their healthcare providers so providing information through alternative channels is important. Educating providers about the importance of the vaccine in pregnancy and providing them with materials in Spanish would be very helpful for increasing vaccination rates. Our study also demonstrated that ads with short, concise facts were attractive to participants who desired more information about the benefits of the COVID-19 vaccine; a web address at the bottom with information in Spanish would be ideal to pair with a social media campaign. Religious messengers in ads were not effective in this study. However, partnering with religious and community leaders should not be discounted on a local level to promote vaccination as they have influence within their own communities. The study also revealed the lack of discussion on vaccine uptake by medical providers.

### 4.4. Strengths and Limitations

A major strength of our study was its focus on an understudied demographic that was both pregnant and Spanish-speaking and was living in rural areas of the Western U.S. Another study strength was the mixed-methods design, which combined quantitative data of the participants’ reactions to social media ads with qualitative data to understand factors influencing their decision to be vaccinated or not to be vaccinated. The ads tested the effect of the messenger and content separately, which improved the study rigor. One limitation of the study is in its generalizability. Our results may not be applicable to Spanish-speaking women in urban areas given that we were specifically targeting rural areas. Additionally, forty percent of the participants preferred not to report their household annual income, which reduced our knowledge of the financial resources of this cohort and ability to assess the impact of income on vaccine uptake. Another limitation was that participants may not have felt comfortable in acknowledging the extent to which they trusted social media for medical information.

## 5. Conclusions

Our study provides critical insight into a highly-vaccine-resistant population, Spanish-speaking pregnant women in the U.S. In this population, we found high trust in the doctor as a messenger and in the recommendation of healthcare providers to become vaccinated. Many women stated that they would have been vaccinated during pregnancy had they received sufficient information about the benefits and risks. Factors such as immigration status had varying influences on vaccination perspectives, while familial and cultural factors often positively influenced views on vaccination. Overall, the respondents preferred social media ads that featured trusted messengers such as doctors along with ads discussing the strong benefits of being vaccinated. These findings help build an evidence base for public health communication campaigns to effectively reach Spanish-speaking pregnant or recently pregnant women and encourage vaccination. As these findings differ somewhat from our prior study enrolling rural English-speaking pregnant and recently pregnant women, who preferred a peer messenger, it is important to investigate diverse sub-populations to understand barriers and facilitators to vaccine uptake [21].

Our results suggest that doctors and other medical providers are powerful messengers for health information in pregnant Spanish-speaking populations in the Western U.S. This knowledge may be leveraged to promote the adoption of a healthy lifestyle, which was negatively impacted by the pandemic [32]. As the Spanish-speaking pregnant population that we studied was predominantly immigrants, a respect for doctors and medical providers to provide health information may translate into other Spanish-speaking pregnant populations in the Global South. Our findings may also help to transform vaccine hesitancy into vaccine acceptance within this Spanish-speaking pregnant population for other vaccinations beyond COVID-19. Understanding knowledge gaps and effective messengers and message will be crucial in understanding how to properly communicate health information to pregnant Spanish-speaking populations in the United States.

## Figures and Tables

**Table 1:** Demographic characteristics of participants.

Characteristic	N (% or IQR)
Median Age	31 (23, 32)
Race and Ethnicity *	
White	1 (3.3)
Hispanic/Latino	29 (96.7)
Declined	1 (3.3)
Pregnancy Status	
Currently Pregnant	13 (56.7)
Pregnant within Last Six Months	17 (43.3)
Number of Children	
None	3 (10.0)
1	11 (36.7)
2–4	16 (53.3)
Marital Status	
Married	13 (43.3)
Single	3 (10.0)
Not Married, Living with Partner	10 (33.3)
Not Married, Not Living with Partner	2 (6.7)
Other	1 (3.3)
Prefer Not to Say	1 (3.3)
Level of Education	
Some High School	4 (13.3)
High School Degree	8 (26.7)
Bachelor’s Degree	8 (26.7)
Graduate Degree	4 (13.3)
Trade School	2 (6.7)
Prefer Not to Say	4 (13.3)
Employment Status	
Employed Full Time	5 (16.7)
Employed Part Time	1 (3.3)
Seeking Opportunities	12 (40.0)
Other	8 (26.7)
Prefer Not to Say	4 (13.3)
Annual Household Income	
<$25,000	9 (30.0)
$25,000–$50,000	6 (20.0)
$50,001–$100,000	2 (6.7)
$100,001–$200,000	1 (3.3)
Prefer Not to Say	12 (40.0)
Religion	
Christian (Catholic)	17 (56.7)
Christian (Any Other Christian Faith)	3 (10.0)
Christian (Protestant)	2 (6.7)
Not Religious	3 (10.0)
Other	2 (6.7)
Prefer Not to Say	3 (10.0)
Political Affiliation	
Very Liberal	6 (20.0)
Slightly Liberal	6 (20.0)
Slightly Conservative	3 (10.0)
Very Conservative	3 (10.0)
Prefer Not to Say	12 (40.0)
Vaccination Status	
Has Received a COVID-19 Vaccine	23 (76.7)
Not Vaccinated	7 (23.3)
Type of COVID-19 Vaccine Received (*n* = 23) **	
Moderna	10 (33.3)
Pfizer	12 (40.0)
Other	1 (3.3)
Number of Boosters Received (*n* = 23)	
None	15 (65.2)
First Booster	3 (13.0)
Second Booster	3 (13.0)
Third–Fourth Booster	2 (8.7)

* Participants could select more than one race or ethnicity. ** Participants could select more than one type of vaccine. IQR = Inter-Quartile Range.

**Table 2:** Number of participants viewing each specific ad.

Messenger	Ad Number	Content	Number of Views
Peer	1	Appeal to Protect	3
	2	Text Heavy	6
	3	Social Proof	7
	4	Negative Outcomes	6
	5	Activation	8
Elder	6	Appeal to Protect	17
	7	Text Heavy	10
	8	Activation	3
Doctor	9	Appeal to Protect	5
	10	Text Heavy	5
	11	Social Proof	13
	12	Negative Outcomes	4
	13	Activation	2
Faith	14	Appeal to Protect	5
	15	Text Heavy	9
	16	Activation	16

Each line represents a specific ad messenger and message content type with the number of times that each ad was viewed in the far-right column.

**Table 3:** Effect of messenger type on ad ratings.

	How Likely to Be Vaccinated after Seeing the Ad			
Predictors	Estimates	Std. Error	Statistic	*p*-Value
(Intercept) ^a^	1.53	0.22	7.00	<0.001
Peer	0.33	0.20	1.69	0.093
Elder	0.23	0.20	1.19	0.238
Faith	0.57	0.20	2.88	0.005

^a^ Doctor was the reference messenger.

**Table 4:** Effect of content type on ad ratings.

	How Likely to Be Vaccinated after Seeing the Ad			
Predictors	Estimates	Std. Error	Statistic	*p*-Value
(Intercept) ^b^	1.60	0.22	7.27	<0.001
Activation	0.50	0.20	2.53	0.013
Social Proof	0.22	0.23	0.98	0.328
Appeal to Protect	0.27	0.20	1.35	0.180
Negative Outcomes	−0.15	0.30	−0.49	0.627

^b^ Negative outcomes were the reference ad content type.

**Table 5:** Themes and sub-themes emerging from the interviews.

Themes
1. The doctor as a trusted messenger and their importance in promoting vaccination: Strong belief in medical training and the expertise of a physician; Inadequate time for counseling by their provider, and access to interpreters cited as a barrier to vaccination; Ancillary staff play important roles building trust with the physician and medical team during pregnancy, especially when the provider does not speak Spanish; Providers had to be prompted to provide COVID-19 vaccine and booster information.
2. Immigration status as an influential factor for vaccine uptake: Fear that immigration status would need to be disclosed; Fear of negative consequences associated with vaccination refusal; Cross-cultural perspective on benefits and risks of vaccination.
3. Generational history of vaccine acceptance through the mother: Memories of being vaccinated with mother; Participants prioritizing vaccinating the next generation/identifying as a good mother through vaccinating children.
4. Participants disliked the use of faith-based social media messages related to vaccine uptake.

## Data Availability

Data are available upon request.

## Supplementary Materials

The following supporting information can be downloaded at: https://www.mdpi.com/article/10.3390/vaccines11111726/s1, Table S1: Interview guide (English and Spanish); Table S2: Thematic codebook; Table S3: Random effects for Table 3; Table S4: Random effects for Table 4; Table S5: Key themes, sub-themes, and quotes; Figure S1: Social Media Ads—Appeal to Protect Content by Messenger; Figure S2: Social Media Ads—Text-Heavy Content by Messenger; Figure S3: Social Media Ads—Social Proof and Informative (Negative Outcomes) Content by Messenger; Figure S4: Social Media Ads: Activation Content by Messenger.
