# Supplementary material for: Factors Influencing COVID-19 Vaccine Uptake among Spanish-Speaking Pregnant People
Source: Vaccines (Basel). 2023 Nov 17;11(11):1726. doi: 10.3390/vaccines11111726 (PMC10674421; doi:10.3390/vaccines11111726)
Supplement: Supplementary file 1 [file vaccines-11-01726-s001.zip › vaccines-2653718-supplementary.pdf]

Supplementary Material

Table of Contents

Table S1. Direct Interview Guide (English and Spanish). 2

Table S2. Thematic Code Book 6

Table S3. Random Effect Estimates of Vaccination Likelihood After Viewing Ads by Messenger 9

Table S4. Random Effect Estimates of Vaccination Likelihood After Viewing Ads by Content Type 10

Table S5. Key themes, sub-themes, and quotes from direct interviews. 11

Figure S1: Social Media Ads: Appeal to Protect Content by Messenger 14

Figure S2. Social Media Ads: Text-Heavy Content by Messenger 15

Figure S3. Social Media Ads: Social Proof and Informative (Negative Outcomes) Content by Messenger 16

Figure S4. Social Media Ads: Activation Content by Messenger 17

Table S1. Direct Interview Guide (English and Spanish).

English Interview Guide

Pregnancy

1. How do you feel about this pregnancy? [Or if it's postpartum: What was your experience with pregnancy like?]
    a. Is this your first pregnancy?
2. Where do you find (or found) support **during** your pregnancy?
    a. **If you've already had the** baby, where do you find support now **after pregnancy** now that your baby is born?
3. How would you describe your relationship with your healthcare provider (doctor or doctor)? Do you trust them? Why yes or why not?
    i. Do your health care provider's (doctor or doctor's) recommendations influence your health decisions? And if they do influence, how do you think they do influence?
    ii. What aspects of your relationship with your doctors or nurses cause you to have more or less trust in your doctors, doctors, or nurses?
    iii. If there is trust, what could they do to further improve that confidence?
    iv. If **there is no** trust, what could they do to build trust in you?

Sources of information

4. Where do you get information to make health decisions during your pregnancy?
5. Have any of the **people or organizations** you consult or ask when you have health concerns mentioned the COVID-19 vaccine?
    a. What information or opinions did you hear from them about COVID-19 vaccines?
6. Did any of **the social media, platforms, or media** you use in your daily life mention the COVID-19 vaccine?
    a. What information or opinions did you hear or see there about COVID-19 vaccines?
7. Who are reliable sources of vaccine information in your **community**? (friends, co-workers, church, support or foster groups)
    a. What have you heard about the COVID-19 vaccine from your community?
        i. *If faith is raised: How is your relationship with this community? What do the leaders of this community do to earn their trust? To what level do you consider yourself religious?*
8. Who are reliable sources of information about the vaccine in your **personal life** (partner, parents, siblings, cousins, uncles)?
    a. What influence do these people have on their health decision-making, their pregnancy, etc.?
9. What sources of information do you **DISTRUST**?
    a. Why do you distrust these sources of information?

Social Media

10. Do you use social media as a source of information related to COVID-19?
    a. What sites/apps do you use? (See demo survey)
    b. What do you use these social networks for? What kind of information do you get in them?
    c. What aspects do you like about these social networks? the videos? the photos? the written text?
    d. Do you trust the information you find on these social networks?

e. If you **rely on** these social networks, which of these social networks do you use specifically to find information about COVID-19 vaccines? Why?<br>f. Are there any of these social networks that you use frequently that you **specifically distrust** to find information about COVID-19 vaccines? Which?
<u>Vaccines and Covid 19</u><br>11. How worried have you been about getting COVID-19?<br>a. How worried were you **before** pregnancy?<br>b. How worried were you **during** pregnancy?<br>c. How worried were you **after** the pregnancy?<br>12. Have you received the COVID-19 vaccine?<br>a. If **you are vaccinated,** what factors influenced your decision to get vaccinated?<br>--What has been **your** experience with the COVID-19 vaccine? How did it feel?<br>b. If **not,** what factors influenced your decision not to get vaccinated?<br>13. What has been **your** experience with other vaccines in general throughout your life? How have you felt about them?<br>a. **If you have** other vaccines, what has motivated you to get other vaccines before?<br>b. **If you** have not had other vaccines, what motivated you not to get other specific vaccines?<br>14. What has been your **child**'s experience with other vaccines?<br>a. If they have other vaccines, what motivated you to get other vaccines before? Why did you decide to give those vaccines to your children?<br>b. If you haven't had other vaccines, what motivated you not to get other specific vaccines?<br>15. Have you received **other vaccines during your pregnancy**?<br>16. Has your perspective on vaccines changed in the past two years since the pandemic began?<br>a. What was your opinion on vaccines **before** the pandemic?<br>b. What was your opinion on vaccines **after** the pandemic?<br>c. ***If you had a change of mind***, has your change of perspective influenced the decisions you've made about other vaccines for your children?<br>17. Do you talk or talk about vaccines in your family? How do you talk to your family about vaccines? What do you say?<br>18. **If you are not vaccinated:** What are the reasons for not getting vaccines? Do your family members think the same way?
<u>Other:</u><br>19. How do you think living in a rural area can affect your access to reliable information about vaccination during pregnancy?<br>20. Do you think a person's immigration status may affect their decision to get a COVID vaccine? How do you think it may affect you?<br>21. Are there more experiences/stories you'd like to share?<br>22. If something comes up even after this interview, do not hesitate to contact us.
<u>After seeing the Creative Ads:</u><br>23. What kind of messages do you think would motivate you, or other pregnant women, to get vaccinated against Covid-19? In other words, what kind of information would you like to see in these types of notices to make an informed decision about getting vaccinated or not?<br>24. Who would you like to be the "messengers" or those who give you information about covid-19 vaccines? Who would you like to see in those ads? (doctors, nurses, friends, family, priests or people from your church, scientists?)

Spanish Interview Guide

<u>El Embarazo</u>

1. ¿Cómo se siente con este embarazo? [O si es posparto: ¿Cómo fue su experiencia con el embarazo?]
    a. ¿Es su primer embarazo?
2. ¿Dónde encuentra (o encontró) apoyo **durante** su embarazo?
    a. **Si ya tuvo al bebé** ¿Dónde encuentra apoyo ahora **después del embarazo** ahora que su bebé ya ha nacido?
3. ¿Cómo describiría su relación con su proveedor de salud (médico o doctor)? ¿Usted confía en ellos? ¿Por qué sí o por qué no?
    i. ¿Las recomendaciones de su proveedor de salud (médico o doctor) influyen en sus decisiones de salud? Y si es que influyen, ¿cómo cree usted que influyen?
    ii. ¿Qué aspectos de la relación con sus médicos o enfermeras provocan que usted tenga más o menos confianza en sus médico, doctores o enfermeras?
    iii. Si es que **hay** confianza ¿Qué podrían hacer para mejorar aún más esa confianza?
    iv. Si es que **no hay** confianza ¿Qué podrían hacer para generar confianza en usted?

<u>Fuentes de información</u>

4. ¿Dónde obtiene información para tomar decisiones de salud durante su embarazo?
5. ¿Alguna de las **personas u organizaciones** a quienes ud consulta o pregunta cuando tiene dudas de salud mencionó la vacuna COVID-19?
    a. ¿Qué información u opiniones escuchó de ellos sobre las vacunas contra el COVID-19?
6. ¿Alguna de las **redes sociales, plataformas, o medios de comunicación** que usted usa en su vida diaria mencionó la vacuna COVID-19?
    a. ¿Qué información u opiniones escuchó o vio ahí sobre las vacunas contra el COVID-19?
7. ¿Quiénes son fuentes confiables de información sobre la vacuna en su **comunidad**? (amistades, compañeros de trabajo, iglesia, grupos de apoyo o acogida)
    a. ¿Qué ha escuchado acerca de la vacuna COVID-19 de su comunidad?
        i. *Si se plantea la fe: ¿Cómo es tu relación con esta comunidad? ¿Qué hacen los líderes de esta comunidad para ganarse su confianza? ¿Hasta qué nivel te consideras religioso?*
8. ¿Quiénes son fuentes confiables de información sobre la vacuna en su **vida personal** (pareja, padres, hermanos, primos, tíos)?
    a. ¿Qué influencia tienen estas personas en su toma de decisiones en salud, en su embarazo, etc.?
9. ¿En qué fuentes de información usted **DESconfía**?
    a. ¿Por qué desconfía de estas fuentes de información?

<u>Redes Sociales</u>

10. ¿Utiliza las redes sociales como fuente de información relacionada con el COVID-19?
    a. ¿Qué sitios/aplicaciones utiliza? (See demo survey)
    b. ¿Para qué usa estas redes sociales? ¿qué tipo de información obtiene en ellas?
    c. ¿Qué aspectos le gustan de estas redes sociales? los videos? las fotos? el texto escrito?
    d. ¿Usted confía en la información que encuentra en estas redes sociales?
    e. Si usted **confía** en estas redes sociales, ¿En cuáles de estas redes sociales que usted usa, **confía** específicamente para encontrar información sobre las vacunas contra el COVID-19? Por qué?

f. ¿Hay alguna de estas redes sociales que usted usa frecuentemente en las que usted **Desconfíe** específicamente para encontrar información sobre las vacunas contra el COVID-19? Cuáles?

Vacunas y Covid 19

11. ¿Qué tan preocupada ha estado por contraer COVID-19?
    a. ¿Qué tan preocupada estaba **antes** del embarazo?
    b. ¿Qué tan preocupada estaba **durante** el embarazo?
    c. ¿Qué tan preocupada estaba **después** el embarazo?
12. ¿Ha recibido la vacuna COVID-19?
    a. Si **sí está vacunada,** ¿qué factores influyeron en su decisión de vacunarse?
       --¿Cuál ha sido **fue su** experiencia con la vacuna contra el COVID-19? ¿Como se sintió?
    b. Si **no,** ¿qué factores influyeron en su decisión de no vacunarse?
13. ¿Cuál ha sido **su** experiencia con otras vacunas en general a lo largo de su vida? Como se ha sentido con ellas?
    a. **Si tiene** otras vacunas ¿Qué la ha motivado a ponerse otras vacunas anteriormente?
    b. **Si no** se ha puesto otras vacunas ¿Qué le ha motivado a no ponerse otras vacunas específicas?
14. ¿Cuál ha sido la experiencia de **sus niños o niñas** con otras vacunas?
    a. Si ellos tienen otras vacunas ¿Qué la motivó a ponerle otras vacunas anteriormente? Por qué decidió poner esas vacunas a sus hijos?
    b. Si no les ha puesto otras vacunas ¿Qué la motivó a no ponerse otras vacunas específicas?
15. ¿Ha recibido **otras vacunas durante su embarazo**?
16. ¿Ha cambiado su perspectiva sobre las vacunas en los últimos dos años desde que comenzó la pandemia?
    a. ¿Cuál era su opinión sobre las vacunas **antes** de la pandemia?
    b. ¿Cuál era su opinión sobre las vacunas **después** de la pandemia?
    c. ***Si tuvo cambio de opinión***, ¿Ha influido su cambio de perspectiva en las decisiones que ha tomado con respecto a otras vacunas para sus hijos?
17. ¿Se habla o conversa sobre las vacunas en su familia? ¿Cómo hablas con tu familia sobre las vacunas? ¿Qué se dice?
18. **Si no está vacunado:** ¿Cuáles son las razones para no recibir las vacunas? ¿Los miembros de su familia piensan de la misma manera?

Otro:

19. ¿Como cree que vivir en un área rural puede afectar el acceso que usted tiene a información confiable sobre la vacunación durante el embarazo?
20. ¿Cree que el estado migratorio de una persona puede afectar su decisión de vacunarse contra el COVID? ¿Cómo cree que puede afectarlo?
21. ¿Hay más experiencias/historias que le gustaría compartir?
22. Si surge algo incluso después de esta entrevista, no dude en comunicarse con nosotros.

Preguntas para despues de ver los Avisos:

23. ¿Qué tipo de mensajes cree usted que la motivarían, o a otras mujeres embarazadas, a vacunarse contra el Covid-19? En otras palabras, ¿Qué tipo de información le gustaría ver en este tipo de avisos para tomar una decisión informada sobre vacunarse o no?
24. ¿Quiénes le gustaría a usted fuesen los "mensajeros" o quienes le entreguen información sobre las vacunas contra el covid-19? ¿A quiénes usted le gustaría ver en esos avisos? (a médicos, enfermeras, amigos, familia, sacerdotes o gente de su iglesia, científicos?)

Table S2. Thematic Code Book

Parent Code	Child Code	Description
**Demographic Variables**	Individual	Refers to individual variables such as age, income, faith, etc.
	Household	Refers to household variables such as number of people in house, HH income, living situation, etc.
	Pregnancy-Related	Refers to variables related to pregnancy, such as gestational diabetes, trimester
	Personality	Refers to references about own personality
	Mental Health	Refers to references about mental health before, during or after pregnancy. Ex. post-partum depression
**Fetus/ Baby**	Perceived Susceptibility	Refers to fetus' or baby's susceptibility to COVID, flu or any vaccine-preventing disease
	Perceived Severity	Refers to the severity of negative impacts to fetus' or baby's health due to a vaccine
	Complacency	Refers to the acknowledgement of susceptibility or severity of COVID, but does not think the risk is high for fetus/ baby
	Perceived Benefits	Refers to the benefits vaccination would have to the fetus' or baby's health
	Motivation	Refers to motivation to get vaccinated relating to the fetus or baby. Ex. to protect baby
**Self**	Perceived Susceptibility	Refers to own susceptibility to or risk of COVID, flu or any vaccine-preventing disease
	Perceived Severity	Refers to the severity of negative impacts to own's health due to a vaccine
	Complacency	Refers to the acknowledgement of susceptibility or severity of COVID, but does not think the risk is high to self
	Perceived Benefits	Refers to the benefits vaccination would have to own's health
	Motivation	Refers to motivation to get vaccinated relating to self. Ex. to protect oneself from disease

**Family/ Network (Includes partner/ spouse, immediate family, friends, community)**	Perceived Susceptibility	Refers to family/ network's susceptibility to COVID, flu or any vaccine-preventing disease
	Perceived Severity	Refers to the severity of negative impacts to family/ network's health due to a vaccine
	Complacency	Refers to the acknowledgement of susceptibility or severity of COVID, but does not think the risk is high to family/ network
	Perceived Benefits	Refers to the benefits vaccination would have to the family/ network's health
	Motivation	Refers to motivation to get vaccinated relating to family/family networks. Ex. to protect family/family network
**Convenience**	Availability	Refers to availability of vaccines in community, doctor's office, etc.
	Affordability	Refers to how affordable vaccines are, or if they're covered by insurance
	Ease of Access to Prenatal Care	Refers to access to prenatal care
	Service Quality	Refers to quality of health care. Can refer to prenatal or vaccine care
	Immigration Status	Refers to the impact of immigration status
	Language	Refers to language as a barrier, or lack of interpretation/ translation services provided
**Cues to Action (Promoting from….)**	Medical Staff/ Provider	Refers to prompting to get, or not get, vaccine from medical care team
	Social Media	Refers to prompting to get, or not get, vaccine from social media
	Partner/ Spouse	Refers to prompting to get, or not get, vaccine from partner/ spouse
	Family Members	Refers to prompting to get, or not get, vaccine from family members
	Friends/ Network/ Community	Refers to prompting to get, or not get, vaccine from friends/ network/ community
**Confidence**	Vaccine Confidence	Refers to belief that the vaccine is safe and effective

	Development Confidence	Refers to belief that the system and people who deliver the vaccine are reliable and competent

Table S3. Random Effect Estimates of Vaccination Likelihood After Viewing Ads by Messenger

Random Effects	Estimates [a]
σ^2 (residual variance)	0.58
τ_{00} (random intercept variance)	0.86
N	30
Observations	120
Marginal R^2	0.028
Conditional R^2	0.608
[a] Doctor was the reference messenger	

Table S4. Random Effect Estimates of Vaccination Likelihood After Viewing Ads by Content Type

**Random Effects**	**Content Type Estimates [b]**
σ^2 (residual variance)	0.59
τ_{00} (random intercept variance)	0.87
N	30
Observations	120
Marginal R^2	0.029
Conditional R^2	0.609
[b] Negative outcomes were the reference ad content type	

Table S5. Key themes, sub-themes, and quotes from direct interviews.

Theme 1. The doctor as a trusted messenger and their importance in promoting vaccination

Strong belief in medical training and professionalism

- "I do trust what health providers tell me. I feel that they are educated people who have experience. They are kept informed… they update their knowledge. And, therefore, I trust in what they tell me."
- "I do trust them. Well, they're educated and apart from that, what they recommend or they tell me, well I do it and yes, it has always helped me."

Inadequate time for vaccine counseling by their provider and access to interpreters cited as a barrier to vaccination

- "Since we are in a rural area, they have trouble keeping their staff. Then their staff is more hurried, they give worse service, so maybe they don't even have time to mention these things to you."
- "So, I trust the doctor, I mean, they should be the first source of information, but yes, they should spend a little more time or if they would provide more information, such as, 'Look, we're going to do these tests. These exams are for this or [ask] is everything okay?' But if you don't ask, they just don't tell you. That would be one area of improvement."
- "She is a very good person, a very good doctor… She only speaks is English, but I feel, well my language is Spanish, right? But I learned a little English, so then she tries to understand me, and I try to make myself understood by her or she provides me an interpreter… [but] I personally feel more comfortable when I talk directly to her."
- "In other clinics, where I got the infusion, there are no staff who spoke Spanish. So it was a little complicated for me. And here with this doctor, because she had a lot of patience and sometimes she also tried to speak Spanish, so if I wanted to feel good being there, because I could understand and they had a lot of patience there. They are very, very friendly."

Ancillary staff play important roles building trust with the patient, especially when the provider did not speak Spanish

- "I think we communicated well. However, of course, if there had been another option of a gynecologist that had been in Spanish, then I had taken it without thinking about it. But since I didn't find any, then, well, I still communicated with him in English, and when I had any doubts about specific words or something specific, that I did not understand him, because I asked the nurse, what this means or what he meant by this?"
- "I asked her [the nurse] a lot … she did a lot of the work that I think the doctor should have done… I probably wouldn't have known 80% of the stuff about my pregnancy and after the baby was born or postpartum. But, there are many people who do not have that resource."

Providers had to be prompted to provide COVID vaccine and booster information

- "There was one of the gynecologists I was seeing and when I told him of my concern about getting the COVID booster, he told me. 'Oh, well, I can't tell you that you have to take it. If you don't want to take it, you don't have to take it'... then I returned to my work and I mentioned it to the doctor I work with and she is the one who convinced me to take the booster".
- "No, they never mentioned any of that [COVID vaccine] to me. What they did mention during the last appointment was one against whooping cough and I soon as they recommended it, I decided to take it, but regarding COVID, they haven't brought it up at all throughout the pregnancy."

• "The first doctor I had, she told me it was an obligation to get the booster, but [after she left] the other doctors no longer asked. They only saw that I had the 2 and I didn't end up getting the booster. And no, they didn't mention it again, if I wanted it."<br>• "[What influences your decision to get vaccinated against COVID during your pregnancy?] Well if they [doctor] tell me, then yes. [If who tells you, your doctor?] Yes, or the social worker. If they tell me, I need to get vaccinated, then I'll say yes."<br>• "[Were you offered a booster during pregnancy?] No, no, he didn't offer it to me. [And when they asked if you had the vaccine, did they give you some information or opinion about the importance of the vaccine?] No, no, they just asked me, 'Are you vaccinated against COVID: yes or no?'"<br>• "Well, so far, I don't feel that I don't [need it]…I know that there are boosters, but honestly, I have never suddenly needed to find out where they are giving them, and they are not even offering them anymore. And I think it's more because of that."<br>• "[They [medical team] haven't offered you any vaccines during your pregnancy?] No, none"<br>• "In fact, they have not told me anything, but I have been thinking about asking for a booster. I've been thinking about getting because I say if, I already have the two, well it doesn't affect me at all to get the booster, right? [And no one at the clinic has mentioned the booster?] At this moment, no."<br>• "I had told them, let me think about it and I will let you know. And well, I forgot. I don't know if it was the pregnancy, life, work, family, ect. And I completely forgot about it. At the next appointment with my doctor, I tried to ask him, and it was really close to my due date, and they said, 'No' Like I had already run out of time [to get the vaccine]."
**Theme 2. Immigration status as an influential factor for vaccine uptake**
• "At first…that's why I think they did as some campaigns around, "Anyone can be vaccinated. It doesn't have a cost". However, when some people had side effects, I do think they were afraid that OK, if you get sick, what's going to happen to your immigration status? If you can't travel to your country or if you can't work, or if you need hospitalization, but you don't have health insurance."<br>• I, think yes, apart from the cost that the vaccine may have, I believe that as an immigrant, sometimes we don't have easy access or the confidence to go to a hospital. A clinic is very expensive…And there is sometimes the fear of going to give your information to the hospital."<br>• "It [immigration status] can affect your decision to be honest… I've seen this in immigration groups because obviously there are a lot on Facebook … where they post their experiences saying, 'I don't want to get vaccinated, but they want to vaccinate me at the [immigration] medical exam, what do I do?' …And everyone says, 'If you want to pass the, the evaluation, then you have to do it.' So they have to be vaccinated by force. Perhaps it will change the perception in which that person who was afraid that by being forced to do it and see that nothing happens, I guess, that already changes her perspective, because that is another way of learning."
**Theme 3. Generational history of vaccine acceptance through maternal figures**
• "Like I'd mentioned, it was simply that once I saw the information, I saw the scientific basis and said, OK, they vaccinate us for everything ever since we are born so then why should I be afraid right now of another vaccine?"<br>• "I'm all for vaccines, I think they're good for you as a person. So, I say, since I have all my vaccines I think [my children] also deserve to have their vaccines to be healthy. Only in that aspect is when I consider my health to influence theirs."

• "I mean, my mom gave me all my vaccines because she loved me, or because she wanted to take care of me. So I guess, I stayed with that, that I have to take care of me so I have to get my shots… My children are all up-to-date on their vaccinations… for me having all your vaccines is the best proof of love that a parent can give to their children. You going to prevent children from getting sick, that children don't have a risk of dying. They should be up-to-date, that is, for me that a child is vaccinated is extremely important."<br>• "My mom tells me that I should never miss any [vaccines] because she already sees that there are many diseases children can get if you don't get a vaccine… I don't know if moms who don't put them, I've always seen it as something important for children. And I do not miss it."<br>• "My grandmother…she was talking to me about vaccines, I think she's a [trusting] source for me, at least very reliable."
**Theme 4. Participants disliked the use of faith-based social media messages related to vaccine uptake**
• "A pastor is going to give this information from a Christian point of view, but if the doctor is based on science and gives me this information, it would be nice"<br>• "I feel that it was the opposite of what happened with the pandemic where many people thought that science was against religion...The religious component of this ad would not have motivated me any more or less to receive the booster because I already have my beliefs, so one thing would not have affected the other"<br>• "It would make me think 'I'm going to put it in God's hands', but it doesn't motivate you to take the vaccine. It's more like trusting God than the vaccine itself"<br>• "I am very glad that the person recommending is a pastor because a lot of times I feel that the Latino community is driven by their religious leaders, so if the pastor is recommending it, I think that not only does it urge them to find support in religion and in their faith, but also in the recommendations of their doctors or what the CDC says"

Figure S1: Social Media Ads: Appeal to Protect Content by Messenger

Peer Messenger
(AD 1)

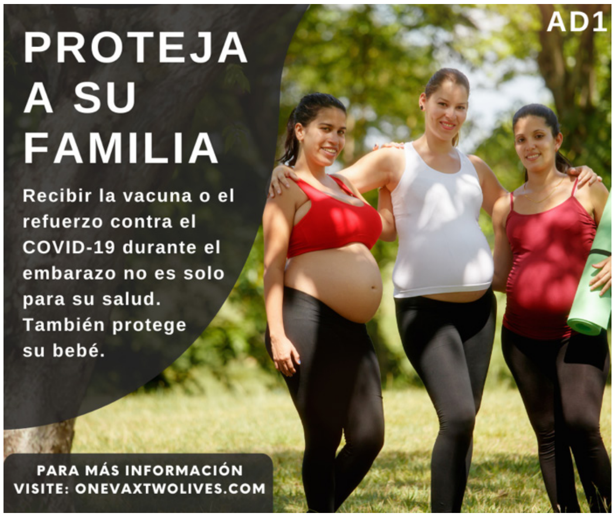

Elder Messenger
(AD 6)

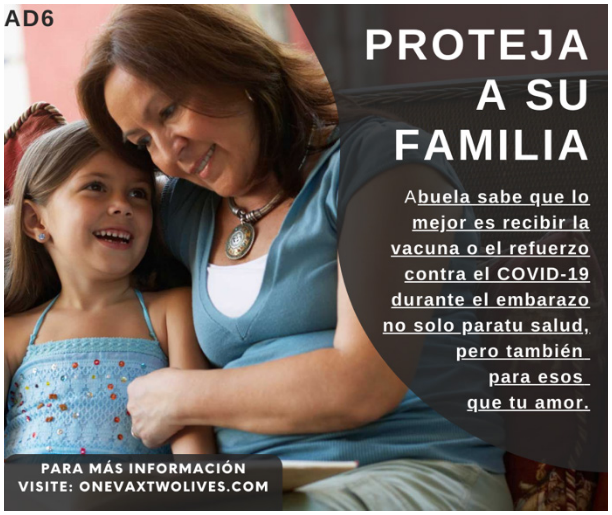

Doctor Messenger
(AD 9)

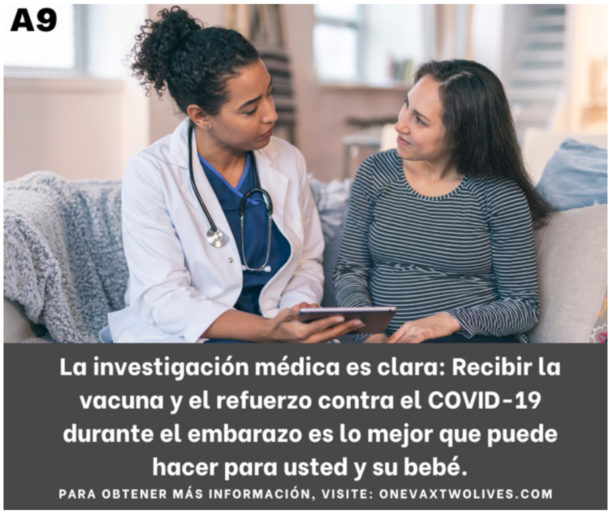

Faith-Based Messenger
(AD 14)

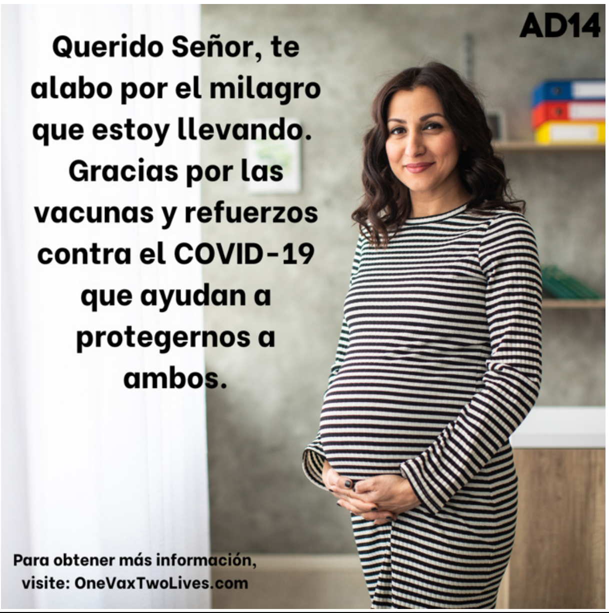

This figure shows the ads tested that appealed to the viewer to protect herself and her fetus from the harms of COVID-19 disease through vaccination. The ads are further broken down by the messenger delivering the message.

Figure S2. Social Media Ads: Text-Heavy Content by Messenger

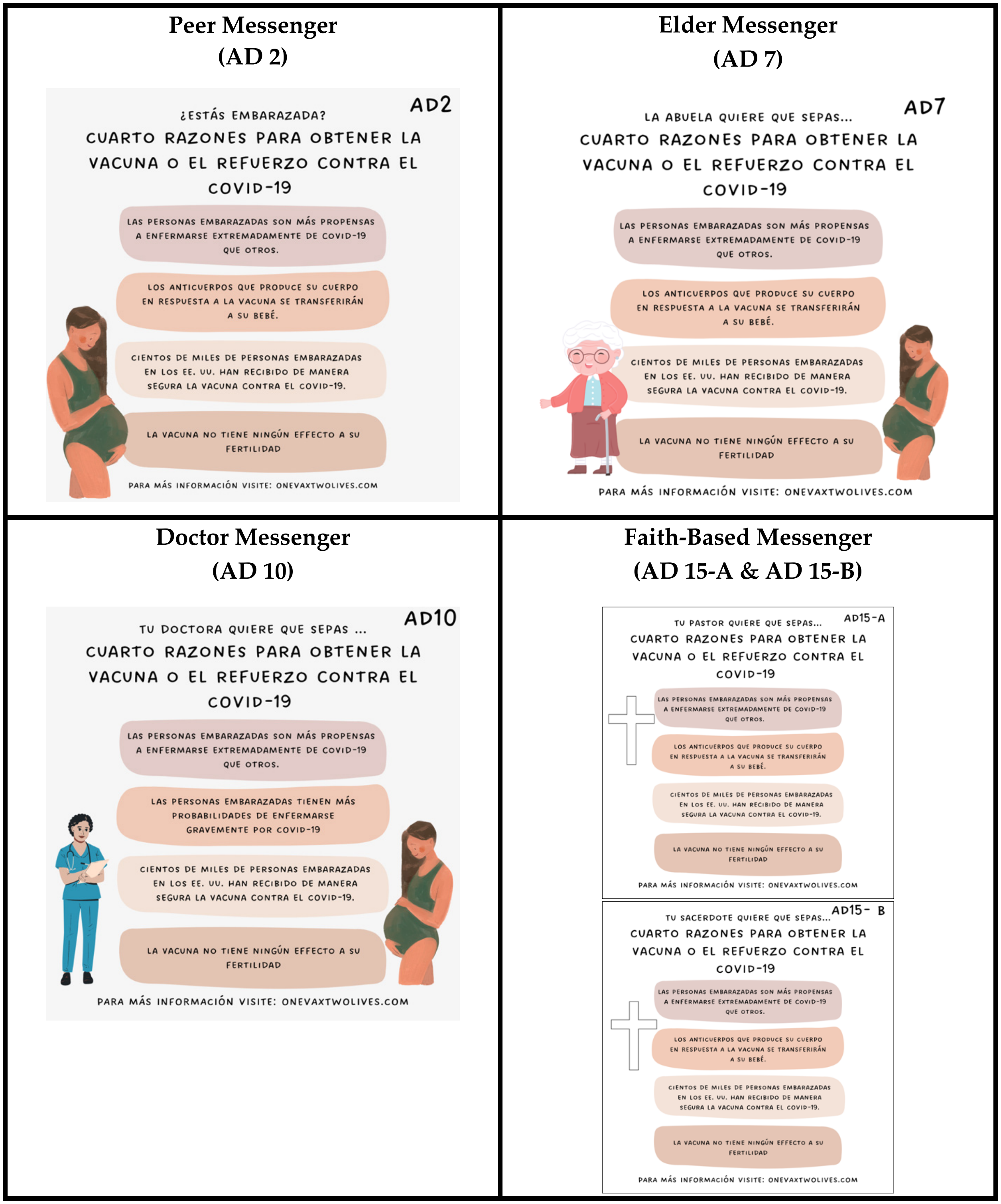

This figure shows ads designed to address pregnant viewers' concerns regarding COVID-19 vaccination by providing text-heavy information. The ads are further broken down by the messenger delivering the message.

Figure S3. Social Media Ads: Social Proof and Informative (Negative Outcomes) Content by Messenger

Content Type: Social Proof

Peer Messenger
(AD 3)

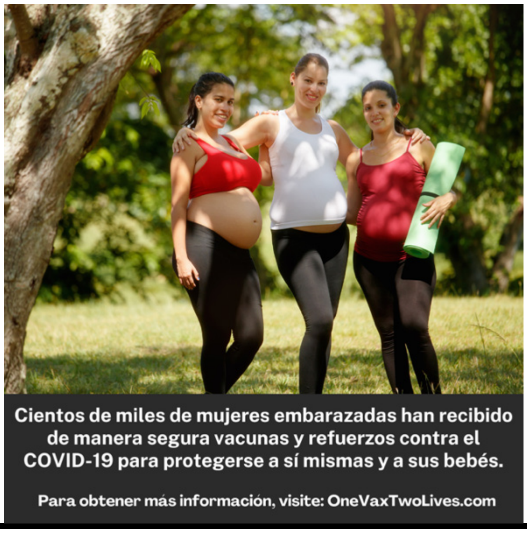

Doctor Messenger
(AD 9)

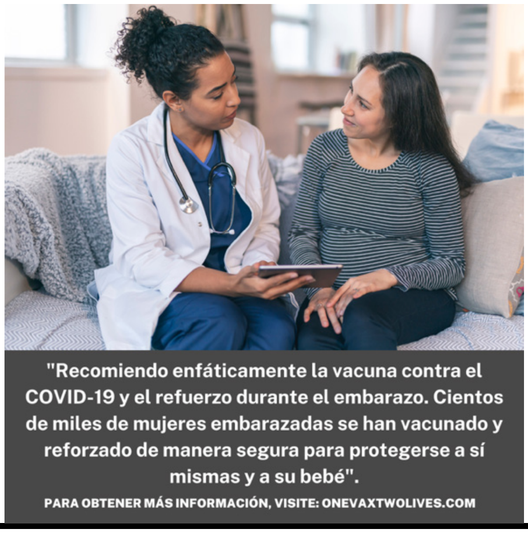

Content Type: Informative (Negative Outcomes)

Peer Messenger
(AD 4)

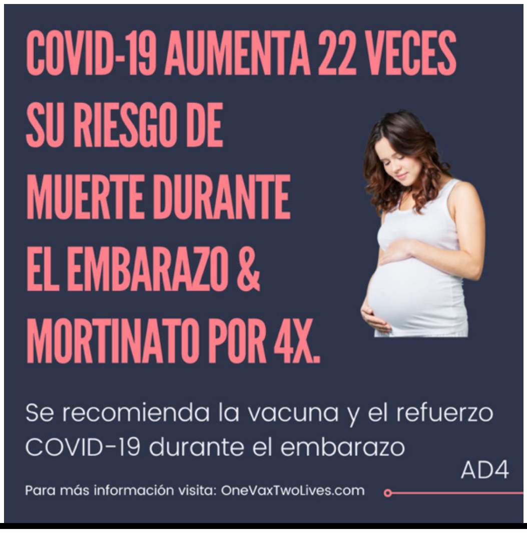

Doctor Messenger
(AD 12)

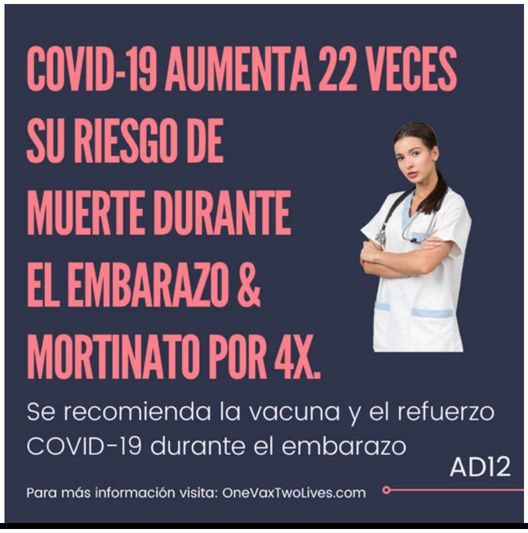

This figure shows the ads designed to appeal to the logic of social proof, where people follow and copy the actions of others (top panel) and the ads designed to provide information regarding the potential negative outcomes for unvaccinated pregnant women (bottom panel). The ads are further broken down by the messenger delivering the message. Not all messengers are shown here as some combinations did not make sense (i.e., negative outcomes provided by a faith leader).

Figure S4. Social Media Ads: Activation Content by Messenger

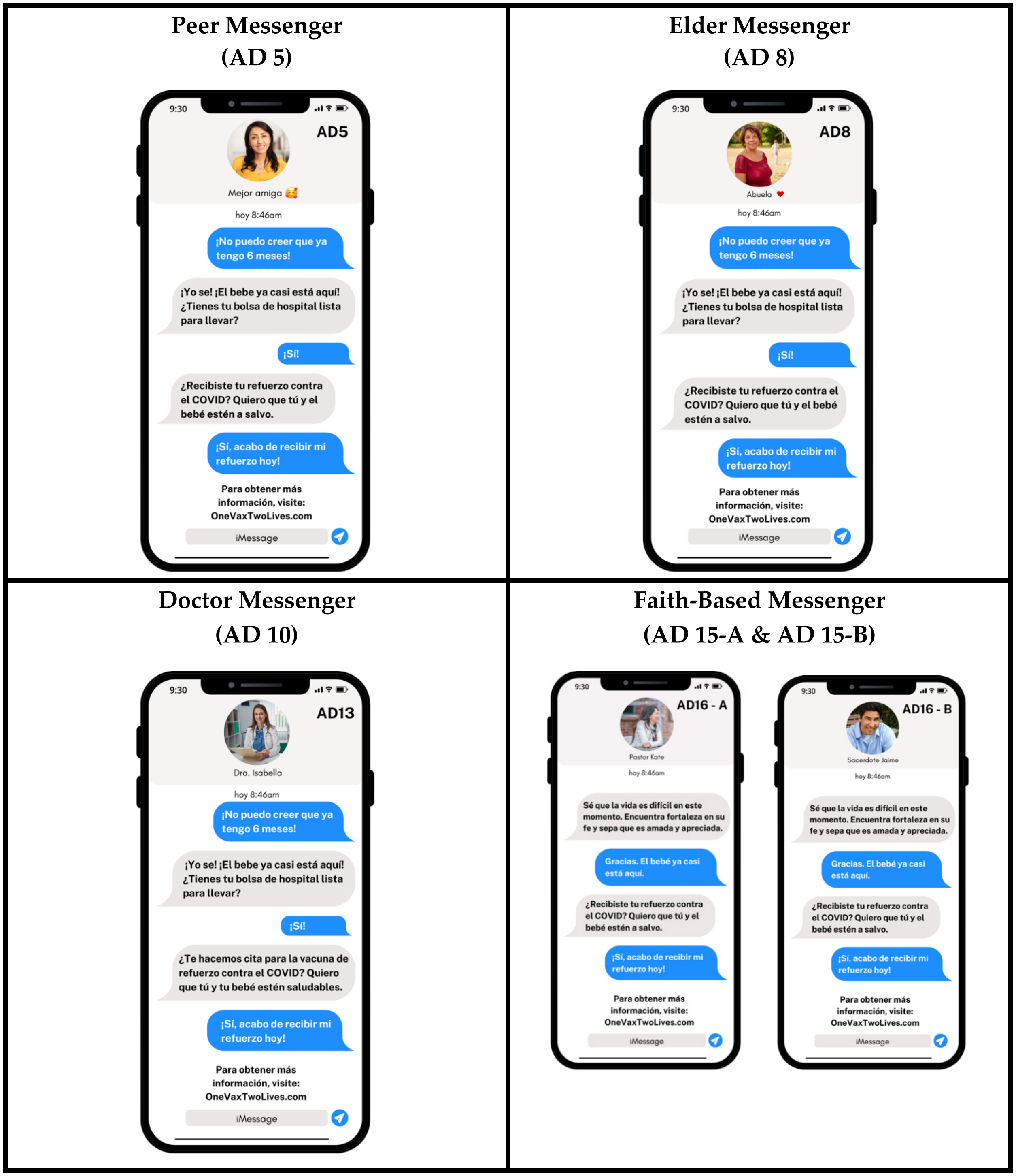

This figure shows the ads designed to activate the pregnant viewer to get the COVID-19 vaccination utilizing activation content. The ads are further broken down by the messenger delivering the message.
